# Erosion: The Probable Causes and Treatment

**Published:** 1882-09

**Authors:** J. J. Bailey


### ARTICLE III.

### *Erosion: The Probable Causes and Treatment.*

BY J. J. BAILEY, L. D. S. EDIN.

[A Paper read before the Students' Society of the National Dental College.]

Mr. President and Gentlemen.—The subject to which I would ask your attention for a few minutes this evening is one which has not, I think, been brought before this Society for a considerable time. It is with little hope of doing justice to the subject that I have undertaken to say a few words concerning Erosion, but rather because I feel sure there are many here who are interested in this question, and the benefit of whose opinions I shall be glad to have.

The disease which we term Erosion, does not seem to have received the attention which its frequency, and the havoc it causes, demand. I think the opinions of nearly all writers on the subject will be found in a paper read by Mr. Coleman before the Internation Medical Congress, 1881, and from which I have made several extracts. The earliest recorded notice is by Hunter, who termed it decay by denudation. After having alluded to the singular appearance these cases present as though filed and highly polished, he concludes as follows, "From its attacking certain teeth rather than others in the same head and a particular part of the tooth, I suspect it to be an original disease of tooth itself, and not to depend upon accident, way in life, constitution, or any particular mangement of the teeth."

Mr. Bell, in a paper entitled "Loss of Enamel and Wear ing Down of Teeth," says, "The groove is perfectly straight and continuous through its whole extent, and is often regular and smooth as if it had been formed by a fine round file and afterwards polished; when the bone has been exposed by this process it sometimes becomes very slightly discoloured, but remains for a long time perfectly hard and sound, and seldom exhibits any appearance of gangrene."

During a discussion at the Odontological Society, in 1870, Mr. Harrison, in the course of some remarks on the subject, said he at one time thought it must be due to certain absorbents in the teeth themselves, not yet discovered; but was obliged to abandon his theory on seeing an artificial lower piece presenting this abnormality. The denture had been carved out of hippopotamus tusk, and had been in the mouth about two years. On coming under Mr. Harrison's notice, he found a number of transverse lines runing from canine to canine. Enquiry elicited the fact that it could not be due to excessive application of the tooth-brush. On seeing this case, he was compelled to abandon his old theory and come to the conclusion that it was, in all probability, produced by some peculiar action of secretion, or of the absorbents, or of both, of the mucous membrane of the lower lip. I have here an upper denture set with natural teeth; and though the majority of the teeth are attacked by caries, I think there are indications of Erosion on several, although some of these seem to have degenerated into true caries.

Mr. Campion is inclined to think it never occurs in necrosed teeth, apparently inclining to the belief that it must be owing to some vital process.

Mr. Salter, in his " Dental Pathology and Surgry," under the heading of "Surface Wear," after alluding to the similarity of Erosion and abrasion, proceeds as follows, " The causes of surface wear may be stated to be predisposing, consisting of an inherit softness of structure which certainly exists in some syphilitic teeth, and probably in other conditions ; and exciting causes, such as molar mastication with incisor teeth, gritty food, a hard tooth brush, certain tooth powders, especially vegetable charcoal, which usually contains particles of silicates of lime and potash. It is probable that in some cases the wearing. is assited by a solvent or softening action of the saliva, but the polish I believe is always occasioned by friction." He seems rather inclined to attribute many of these cases to syphilis, especially those where the cutting edges of incisor teeth are reduced.

Dr. Magitot considers these clean and polished notches as nothing else than caries which has passed into the condition of spontaneous cure or dry caries.

Mr. Coleman has arrived at the conclusion," That the main element is friction, but that the condition which permits the loss of substance thereby, is favoured by a change or degeneracy in the tooth itself." He suggest that change may be owing to fatty degeneration of the contents of the dentinal tubuli and the formation of a fatty acid ; or, as an alteration, he surmises it might result from lessening of that power of resistance vaguely termed vital force. But, if the latter, one would expect to see it in dead teeth more than in living ones, whereas he leans to Mr. Campion's view that it does occur in necrossed teeth, which teeth must have lost their power of resistance. With regard to the agencies which furnish the friction, he says he has never seen a case in which it could not be imputed to either tooth-pick, brush, lips, tongue or saliva.

I have endeavoured to give, briefly, the opinions of several authorities as to the cause of Erosion; but, I imagine, with the result that, though we may believe the old adage, "In a multitude of councellors there is wisdom," we must have come to the conclusion that thier wisdom is in this case *slightly* confusing.

I have purposely left till last the opinion of one great authority—Mr. Tomes. After describing the disease, he sums up as follows, "Absorption cannot be called into account for the removal of the tooth-substance, for it often takes place at spots remote from any structure capable of developing an absorbent organ, and it seems that we must fall back upon the idea that it is an example of chemical solution. But whence the solvent comes, and why the affected surfaces are not the site of ordinary caries, are question which remain unsolved, though it seems probable that mucous, by fermentation, or affording a nucleus for fermentation, may provide an acid solvent.

If I might offer an opinion of my own on the subject, I should feel inclined to accept Mr. Tomes's theory, partly because I may possibly have found a solution of his question as to why the attacked parts are not the seat of caries. Before, however, I ask your consideration of this idea, I shall just remark on one or two particular appearances which these cases present. I do not intend to trouble you with a minute description of the disease, as that is pretty well known to all of us. I think all observers in describing the disease have agreed that these depressions may have sharp, well-defined, or even overhanging edges; the whole circumference may slope off to the surface; but there is one characteristic which has not I think, been remarked. It is that we may have sharp edges, but that they never extend the whole circumference, there will always be found two points, and these opposite points in which the cavity slopes off to the surface of the tooth. In other words, whatever be the shape of the cavity in one direction, sharp edges or sloping, in the other it will always be so formed that instrument or point may be passed from one side across the cavity and emerge at the other side without meeting with any obstruction.

We have seen that Mr. Coleman considers the prime agent as friction, but willing to attribute it to quite a number of causes, viz., tongue, lips, tooth-pick, brush, saliva, etc. Now it seems difficult to think that a disease so unform in its characteristics should be directly caused by so many agencies. I have myself thought we must try to find some cause to which we can always attribute this affection. We know the tooth-brush cannot produce it in every case, though it is undoubtedly often an important factor; neither can the lips, tongue, etc. Mr. Coleman alludes to the action of the saliva, and, in support of that theory, mentions the wearing away of stones by the continual dropping of water.

For some little time I have been observing several cases in the hope that I might possible find a clue. I have not, in the somewhat limited period, seen a very large number of cases; but, from what I have seen, I am inclined to think that Erosion is caused by the mechanical action of the fluids in the mouth. I would give the lips, tongue, etc., a secondary position considering that they cheifly act as motive powers to the fluids. There are one or two reasons why think this to be the case.

In the first place, all cases of Erosion are in a position subject to the action of the oral currents; for the fluids may take such varying directions in different mouths, and even in the same mouth. We know what a small thing is sufficient to vary the current of a running stream, and in like manner a small cause as a slightly projecting tooth may be quite enough to determine the direction the fluids may take. An instance may be found, in say, an upper central incisor, where Erosion has occurred at the neck of the tooth in front. As the denuding process encroaches on the tooth and gets deeper, we may see the lateral extremities take one or two courses; it may take on the form of caries, in which case we shall probably find that the teeth are crowded together so that no fluid could possibly pass between them; or the Erosion may proceed round the side of the tooth, though such tooth will, I think in the majority of cases, be natually narrowed at the neck, and it will be found that there was previously a slight space through which liquid could pass. In some cases the tooth-pick may have helped the process.

Secondly, the characteristic shape of the cavity is such that it will always form a channel along which fluid can passed unobstructed.

Thirdly, though all cases can be accounted for by this agency, there are certain cases which I can account for in no other way. These are some of the reasons why I incline to this view.

Though I think all this to be the mechanical cause, I of course rely upon the idea that there is a predisposing cause though it does not necessarily produce Erosion. I am inclined to think that this is the same that often precedes caries. I imagine there must be some secretion which has the power of softening and dissolving the tooth-substance, the result of which may be either Erosion or Caries. Now what is it that determines which the sequel shall be the former or the latter? Think this entirely depends on whether the attacked part of the tooth is under one or other of certain conditions. If the part is under such conditions that the dissolved substance can be easily and frequently removed, and that by liquids always moving in certain directions, then I apprehend we shall find Erosion will supervene. If, however, the part is in any way protected from exposure to cleansing influences, then the dissolved material will remain and form a nucleus of decay, in which fermentation, with its attendent leptothrix, etc., will soon appear. In short, we shall have all the appearances of caries.

We now come to the question, Why do we see the teeth in one mouth subject to Erosion, and in another mouth the corresponding teeth attacked by caries? The position of the teeth and all natural conditions may apparently be alike in both. I think the deciding point depends upon the extent to which the self-cleansing agencies are at work. We all know what a vast difference there is in the cleansing power of our patients. We might give some glass after glassful of water to rinse their mouths with and they would then have hardly removed any foreign matter which might be on the teeth; while other patients' mouths wou'ld be infinitely cleaner after rising with one mouthful of water. In the former case I do not think we should find Erosion; and, with one exception, I cannot remember having seen any in such a mouth; indeed, since my attention was drawn to the subject, I certainly have not met with it, while, on the contrary, caries has been prevalent enough. Again, in *all* cases of Erosion which have come under my notice, the patients have used the oral muscles to a considerable extent the one exception, before alluded to being that of a molar in rather a prominent position, and, on inquiry, I learned that the patient used a considerable amout of force with the brush. I have frequently and purposely in such cases covered the teeth with a foreign substance, such as pumice powder, having forced it well between the teeth, both those in which Erosion was still progressing, and those in which the contour of the teeth had been restored. Having done this, I have allowed the patient just one mouthful only of water to rinse away the material. I have then examined the mouth, and have invariably found it thoroughly clean,—almost every particle, in some cases every atom of powder having been removed. I have taken numerous opportunities of testing this, and the result has always been the same, the patient having made considerable use of the muscles of the mouth. In polishing fillings for patients not subject to Erosion I have often had the greatest difficulty to remove the polishing material by mere rinsing; they apparently have no idea of moving the muscles of the mouth, but just let the water roll round and out again. How far this may be the result of diffierent temperaments I cannot say, but think that may possibly have some governing influence.

To recapitulate, it seems to me that we may consider Erosion in its first stage to be nothing more or less than the begining of caries; in fact, a dissolving of the tooth-substance which, however, never gets beyond the initial stage, as the materials are removed almost as constantly and continually as they are dissolved. Let this intermittent but frequent removal of the acid solutions cease, and I think we shall find caries will soon appear. We often see teeth attacked by Erosion, and adjoining the denudation we also perceive caries. Though there is a mared contrast between the two, yet the one often merges imperceptibly into the other.

We now come to the treatment of this mysterious affection. This must, until we know the cause, be, to a certain extent, empirical. It should be directed to restoring the tooth to its former contour, thus obliterating the friction channel. Mr. Coleman says he has found the constant application of ammonia, or one of its compounds, if persevered in, to arrest the disease. He use sal-volatile, applied three or four times a day, arguing that the alkilinity of the ammonia may neutralise the acid conditions of the contents of the dentinal tubuli; thus removing the predisposing cause. Other writers recommend the use of silver, but this gives a black unsightly appearance, though, I believe, it is effectual in the majority of cases. Chloride of zinc is also suggested.

Many cases seem to be particularly suitable for gold, providing it is thoroughly well done and polished, care being taken that not the slightest edge is left. But in proportion as the saving power of a gold filling seems to justify its use we often find its unslightly appearance would considerably nullify its advantages. Hence we must frequently have recourse to some other material. The various amalgrams and white cements all give way in these positions, but we have one other substance to fall back upon, and that is gutta-percha. I have known cavities carefully prepared and filled with this to last a long time and give satisfaction to both patient and operator.

In conclusion, while thanking you for the attention you have given me, I cannot but be aware of the many imperfections which exist in the paper; however, I trust that what I have said may have been sufficiently interesting to prove a subject for discussion.—*London Dental Record.*

